# Prehabilitation of surgical patients: a bibliometric analysis from 2005 to 2023

**DOI:** 10.1186/s13741-024-00410-x

**Published:** 2024-05-31

**Authors:** Wei Ma, Yijun Liu, Jin Liu, Yanhua Qiu, Yunxia Zuo

**Affiliations:** 1 Department of Anesthesiology, West China Hospital, Sichuan University, Chengdu, Sichuan People’s Republic of China; 2 The Research Units of West China (2018RU012), Chinese Academy of Medical Sciences, West China Hospital, Sichuan University, Chengdu, Sichuan People’s Republic of China

**Keywords:** Prehabilitation, Bibliometric, Surgery, Perioperative

## Abstract

**Background:**

Good preoperative conditions help patients to counteract surgical injury. Prehabilitation is a multimodal preoperative management strategy, including physical, nutritional, psychological, and other interventions, which can improve the functional reserve of patients and enhance postoperative recovery. The purpose of this study is to show the evolution trend and future directions of research related to the prehabilitation of surgical patients.

**Methods:**

The global literature regarding prehabilitation was identified from The Web of Science Core Collection database. Bibliometric methods of the Bibliometrix package of R (version 4.2.1) and VOSviewer were used to analyze publication trends, cooperative networks, study themes, and co-citation relationships in the field.

**Results:**

A total of 638 publications were included and the number of publications increased rapidly since 2016, with an average annual growth rate of 41.0%. “Annals of Surgery”, “British Journal of Surgery” and “British Journal of Anesthesia” were the most cited journals. Experts from the USA, Canada, the UK, and the Netherlands contributed the most in this field, and an initial cooperative network among different countries and clinical teams was formed. Malnutrition, older patients, frailty, and high-risk patients were the hotspots of recent studies. However, among the top 10 cited articles, the clinical effects of prehabilitation were conflicting.

**Conclusion:**

This bibliometric review summarized the most influential publications as well as the publication trends and clarified the progress and future directions of prehabilitation**,** which could serve as a guide for developing evidence-based practices.

**Supplementary Information:**

The online version contains supplementary material available at 10.1186/s13741-024-00410-x.

## Introduction

With more than 313 million surgeries performed each year globally (Weiser et al. 2015), the quality of postoperative recovery is a major concern for patients, their families, and physicians, while also placing a heavy burden on healthcare systems. The stress response caused by surgical injury can have a serious influence on the internal environment of patients, further affecting the quality of postoperative recovery. In addition to intraoperative and postoperative management, the preoperative healthy state of the patient determines their ability to withstand the perioperative stress (Cusack and Buggy 2020; Gillis et al. 2022). Preoperative malnutrition can increase the risk of perioperative complications by 3 times and mortality by 5 times (Wischmeyer et al. 2018). Poor preoperative aerobic capacity is also an independent risk factor for postoperative morbidity and mortality in patients undergoing intra-abdominal surgery (Moran et al. 2016a). Moreover, preoperative adverse moods, such as anxiety and depression, can significantly increase the incidence of serious postoperative complications and prolong the length of hospital stay (Williams et al. 2013; Orri et al. 2015).

Twenty years ago, the concept of prehabilitation was firstly introduced into the medical field by Topp et al. (2002). Later, prehabilitation was defined as a bundle of evidence-based elements for different preoperative unhealthy states (Carli 2020), including nutritional interventions, exercise training, psychological counseling, etc. The goal of prehabilitation is to increase the functional reserve of patients before surgery, enhance postoperative recovery, and improve quality of life. At present, enhanced recovery after surgery (ERAS) is a hot spot in perioperative practice, and rehabilitation can be regarded as the “first battle” of ERAS. With the growing proportion of elderly, cancer, and frail patients, prehabilitation has received increasing attention in the ERAS management strategy (Kehlet 2021).

Bibliometrics is a method to examine publications in a specific academic field. It quantifies both knowledge structure and emerging research trends through main bibliometric techniques of visualization and clustering which can reveal the characteristics of publications, such as countries, journals, authors, and institutions with outstanding contributions, frequently used keywords, and cooperation networks between countries, institutions, and authors (Donthu et al. 2021). This paper aims to analyze the evolution trend and provide future directions of research related to the prehabilitation of surgical patients through bibliometrics.

## Methods

### Literature search

This study chose the Web of Science Core Collection (WOSCC) as the data source. The search scope was restricted to the Science Citation Index Expanded (SCI-expanded) and Social Sciences Citation Index (SSCI). According to the purpose of the study, the search formula was (TS = (prehabilita* OR pre-habilita*)) AND (TS = (surg* OR operati* OR perioperati* OR preoperati* OR The pre-operati*)). The search ended on December 1, 2023, with no language restrictions.

### Literature screening

We firstly screened the article types, excluding editorial material, book chapters, letters, and meeting abstracts. After that, the content of each article was manually reviewed to eliminate study protocols, duplicates, and unrelated literature. We analyzed prehabilitation as a separate research area, and studies on multimodal ERAS strategies involved in prehabilitation were excluded. Finally, the synonyms in the extracted data were merged by manual checking, including authors’ names, institutions, keywords, etc.

### Data analysis

Two main tools, the Bibliometrix package of R (version 4.2.1) (Derviş 2019) and VOSviewer (Van Eck and Waltman 2010) were used in this study for bibliometric analysis. The former was used for realizing the visualization of quantitative data, mainly for (1) calculating the number of publications in different years, countries, institutions, journals, and authors; (2) determining the popular keywords and their frequencies; (3) calculating the frequency of cooperation between countries; and (4) analyzing co-citation relationships. The latter was a visualization of a bibliometric networks tool, which could realize cluster analysis. VOSviewer was used to present (1) the collaborative network of research institutions and keywords, and (2) the co-citation network of journals and references. Another online tool (https://charticulator.com/) was used for visualization of international cooperation between countries.

## Results

A total of 1584 studies were retrieved, the screening flow chart was shown in Supplementary Figure S1. After being assessed by full text, 638 articles on the prehabilitation of surgical patients were included, including 427 articles and 211 reviews. Among them, 621 articles (97.3%) were published in English, 11 in German, 3 in French and 3 in Spanish.

### Publication summary

**Fig. 1:**
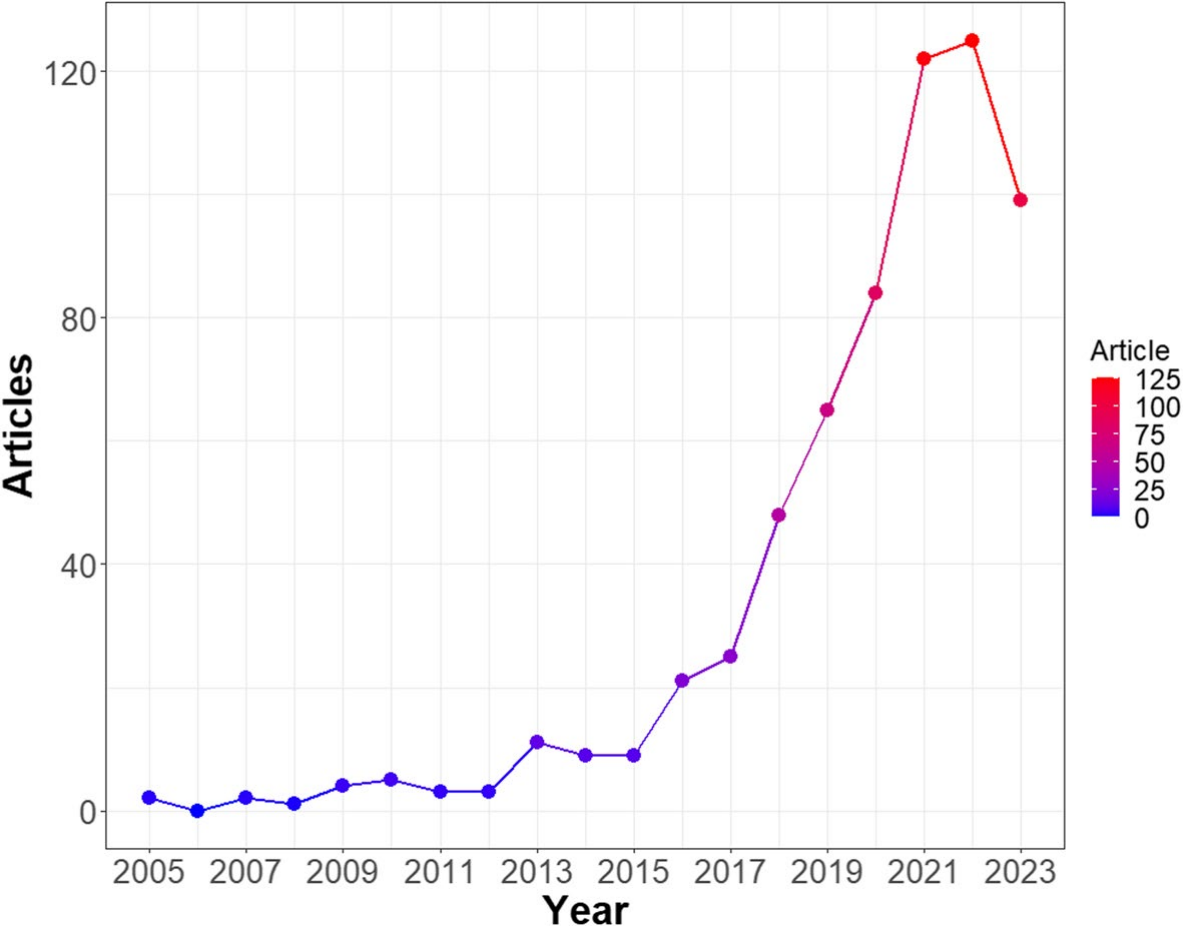
Annual publication counts on prehabilitation of surgical patients during 2005–2023

Figure 1 illustrated the number and trend of the annual publications on prehabilitation of surgical patients from 2005 to 2023. According to the trend, the progress of this field could be roughly divided into three stages: (1) From 2005 to 2012, the number of publications each year was less than 5, indicating that research in this field was in its infancy and had not received attention. (2) From 2013 to 2015, the annual number of publications was slightly higher than before, approximately 10 per year, which formed the second platform period. (3) From 2016 to 2022, the number of articles increased exponentially at an average rate of 41.0%, from 21 articles in 2016 to 125 articles in 2022.

### Cooperative network analysis

**Fig. 2:**
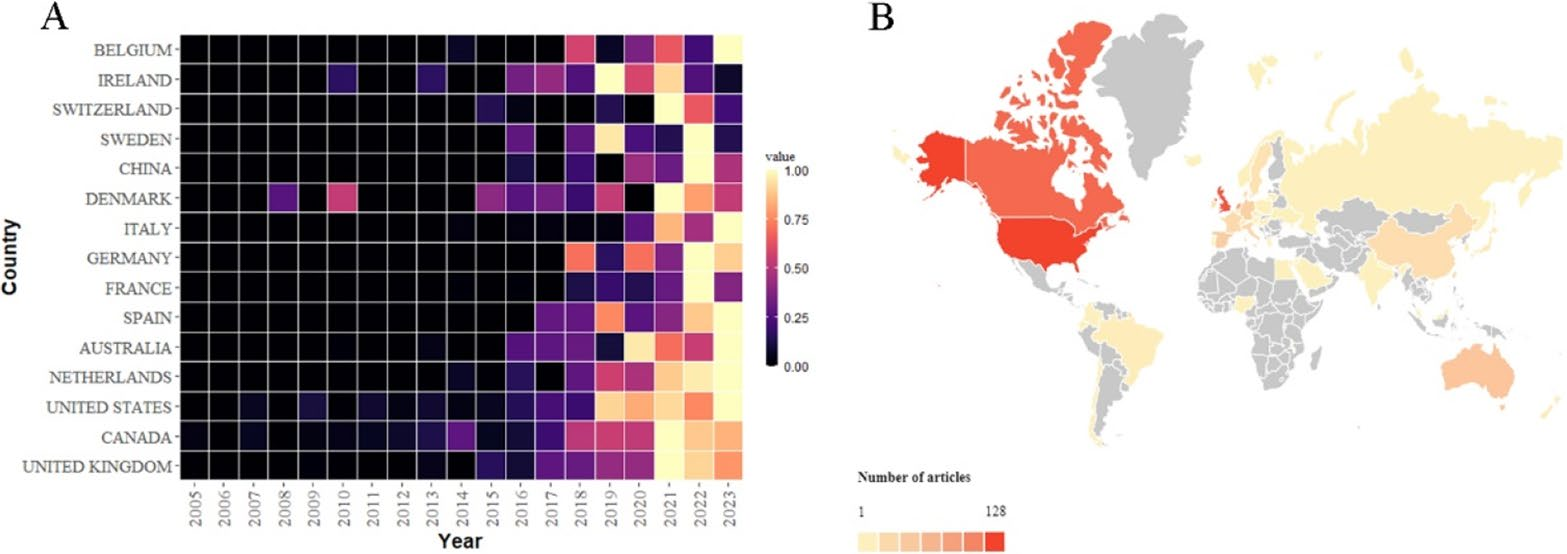
**A** Distribution of publications over time for the top 15 countries with most publications. **B** World map of publication counts

Figure 2A showed the distribution of publications over time for the top 15 countries. Denmark, the USA, and Ireland were among the first countries to conduct prehabilitation studies. The UK and Canada joined them later. However overall, enthusiasm was low in the early stage. Since 2016, especially in the past 3 years, many studies have been conducted by various countries, and prehabilitation has attracted the attention of the global medical community. Countries in North America and Europe led the world in the number of publications in this field (Fig. 2B). Scholars from the United States published the most articles (128 publications), followed by the UK (122 publications), Canada (114 publications), and the Netherlands (82 publications). Publications from other countries were less than 50. Among the top 20 countries with most publications, there was a very close collaboration between them. Cooperation between countries was represented by lines and thicker lines indicate more frequent collaboration (Supplementary Figure S2). Most of the cooperation occurred between European and American countries, with the largest cooperation frequency between Canada and the USA, followed by Canada and Italy, the UK and Australia, and the UK and the USA.

**Fig. 3:**
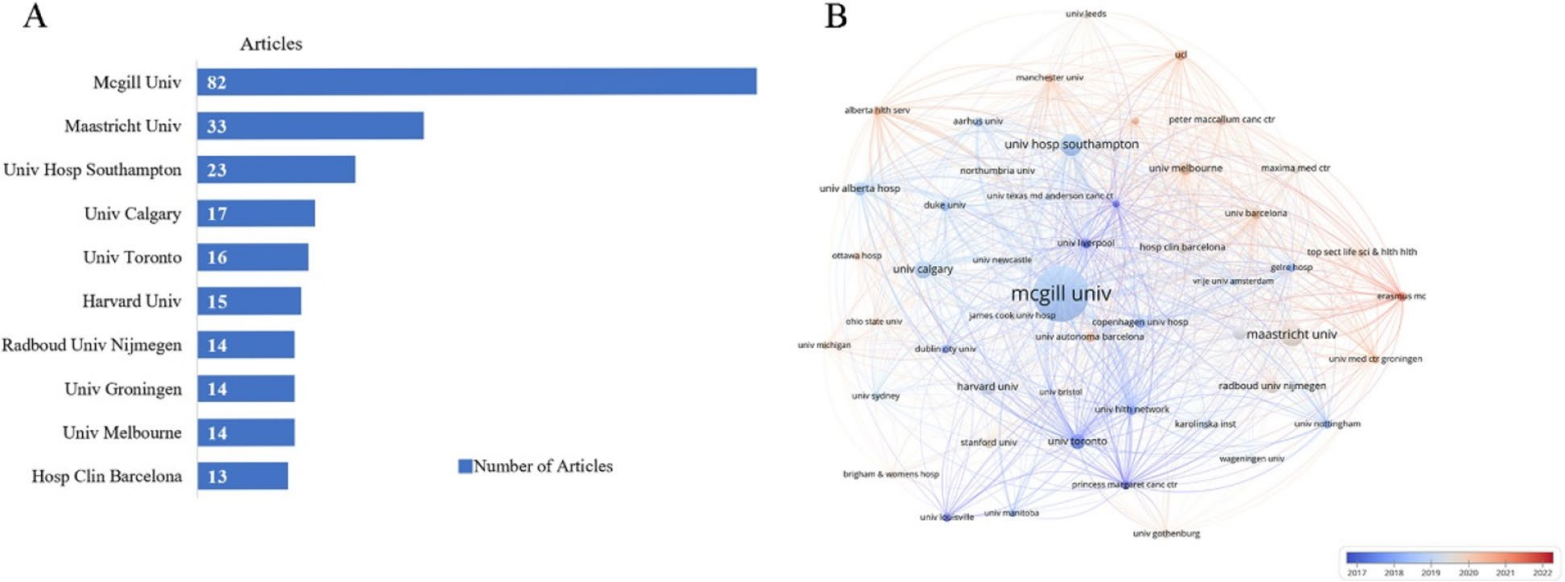
**A** Top 10 productive institutions. **B** Overlay network of co-authorship analysis of the top 50 institutions

A total of 926 institutions contributed to the study of prehabilitation. Figure 3A showed that the 10 most productive institutions came from Canada, the UK, the Netherlands, the USA, Australia, and Spain. In the overlay network of co-authorship analysis of the top 50 institutions, the size of the node reflected the number of publications by institution, The lines between nodes illustrated the collaboration between different institutions, and the colors indicated the average commencement year of publications for each institution (Fig. 3B). Researchers at the University of Toronto, the Princess Margaret Cancer Centre, and the University of Liverpool were pioneers in the field of prehabilitation. In contrast, researchers at Erasmus University and University College London have conducted more recent studies in this field.

A total of 3348 authors published articles on the prehabilitation of surgical patients. Table 1 lists the top 10 authors in terms of publications. Francesco Carli was the most productive author, who had published 67 articles. The G-index is an index obtained by a comprehensive consideration of the number of publications and the citation frequency of articles, which can more accurately reflect the influence of an author in a specific field. The higher the index, the greater the influence of the author. Francesco Carli, Chelsia Gillis, Rashami Awasthi, and Enrico M. Minnella from McGill University are the four scholars with the highest G-index. In the density map drawn based on the number of publications of the authors (Supplementary Figure S3), the deeper the color, the more publications the author has. A productive collaborative group was formed around the above four high-impact authors.

**Table 1:** Top 10 authors in terms of publication counts

Author	Articles	Citations	Average citations	G-index
Francesco Carli	67	3964	59.2	64
Enrico M. Minnella	27	1507	55.8	24
Chelsia Gillis	25	1549	62.0	27
Rashami Awasthi	25	2140	85.6	26
Celena Scheede-Bergdahl	19	1149	60.5	20
Vanessa Ferreira	13	297	22.8	13
Bart C. Bongers	13	270	20.8	16
Malcolm A. West	12	446	37.2	11
Michael P.W. Grocott	12	725	60.4	12
Sandy Jack	11	813	73.9	11

A total of 263 journals published articles on the prehabilitation of surgical patients. Table 2 shows the top 10 journals with the most publications and their impact factors. Half of the top 10 journals came from the UK and four came from the USA, mainly in the fields of surgery, anesthesiology, and oncology. Journal Citation Reports (JCR) could reflect the quality and professional influence of journals. All of the top 10 journals were classified into JCR-Q1 or Q2, indicating that prehabilitation of surgical patients was a topic worthy of discussion.

**Table 2:** Top 10 journals with the most publication counts

Journals	Articles	Country	Impact factor	JCR category
European Journal of Surgical Oncology	25	UK	3.8	Q2
Supportive Care in Cancer	25	USA	3.1	Q2
Perioperative Medicine	14	UK	2.6	Q2
Anaesthesia	11	UK	10.7	Q1
PLoS One	10	USA	3.7	Q2
British Journal of Anaesthesia	9	UK	9.8	Q1
Cancers	9	Switzerland	5.2	Q2
Annals of Surgery	8	USA	10.1	Q1
International Journal of Surgery	8	UK	15.3	Q1
Journal of Gastrointestinal Surgery	8	USA	3.2	Q1

*JCR* journal citation reports, *UK* the United Kingdom, *USA* the United States of America

### Study theme analysis

**Fig. 4:**
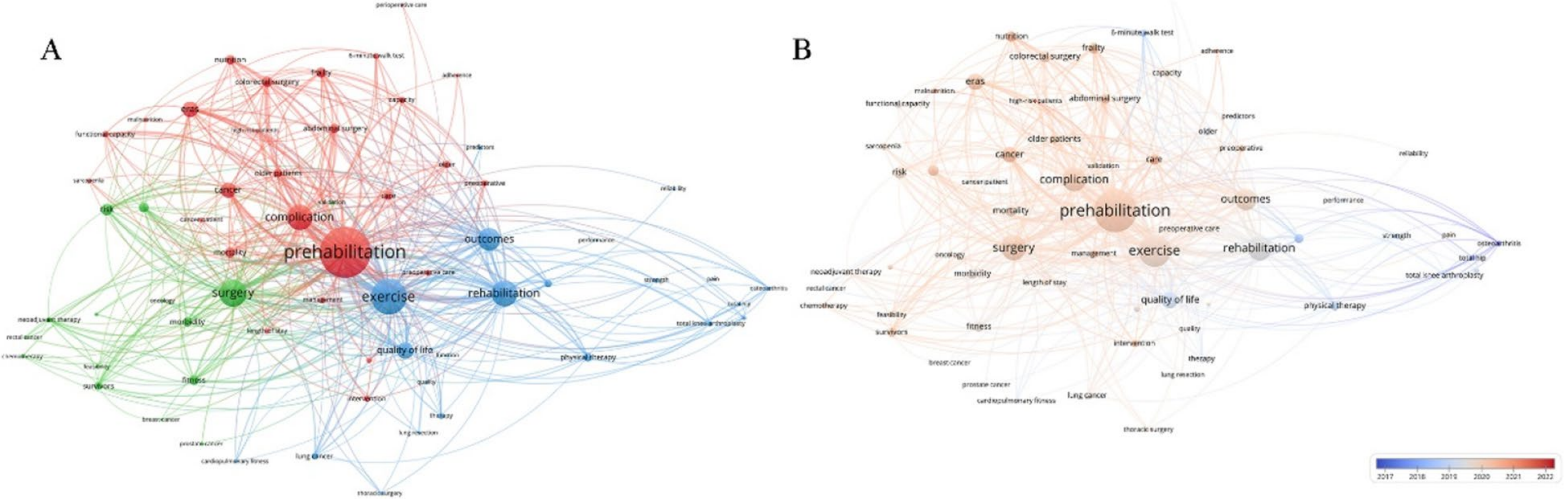
**A** Network visualization of keywords. **B** Overlay network of keywords

Exploring the occurrence frequency of keywords and co-occurrence analysis can quickly focus on research hotspots and reflect the changing trend of research hotspots. Out of 1742 keywords, the lowest frequency limit of occurrence was set to 16 according to Price’s law. A total of 72 keywords were included in the analysis. “Prehabilitation” was the most frequently used keyword (frequency = 428), followed by “exercise” (frequency = 286) and “surgery” (frequency = 207). “Colorectal cancer”, “colorectal surgery” and “abdominal surgery” exhibited a notably higher frequency than other diseases and types of surgery, indicating that many prehabilitation-related studies had been carried out in such patients. Figure 4A showed the results of the cluster analysis generated by VOSviewer. The size of the node reflected the frequency of the keyword, while the line between the two nodes reflected the co-occurrence. All keywords were grouped into 3 clusters that roughly reflect the main themes in the research field. The red cluster mainly focused on the terms “prehabilitation”, “ERAS” and “complication”. The green cluster focused on surgical-related conditions, which included “surgery”, “colorectal cancer”, and “morbidity”. The main keywords in the blue cluster were “rehabilitation”, “outcomes”, and “quality of life”. In addition, terms related to “exercise” were included in the blue cluster. In general, the most prominent nodes in Fig. 4A summarized the connotation of prehabilitation: implementation before surgical procedures with the purpose of improving postoperative recovery and outcomes of the patients. The overlay visualization of keywords and average time of appearance showed the changes in research hotspots over time and pointed out the research trends in the future. In Fig. 4B, keywords appearing earlier were shown in blue, while orange represented keywords appearing more recently. “Total knee arthroplasty”, “osteoarthritis”, and “physical therapy” were topics of early attention. In contrast, “malnutrition”, “older patients”, “frailty”, and “high-risk patients” have recently received considerable attention from researchers in the field.

### Co-citation analysis

**Fig. 5:**
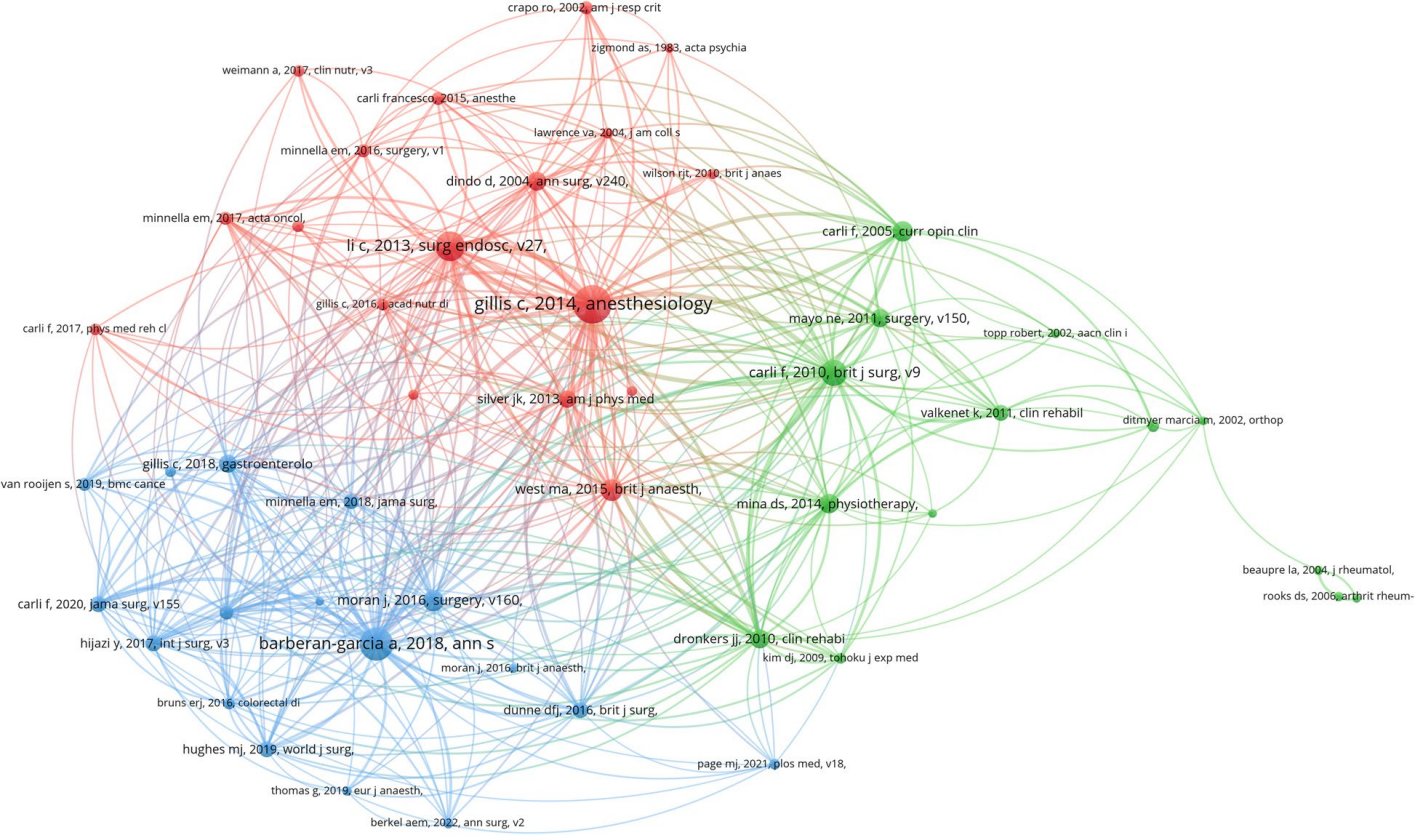
Co-citation network of references

Co-citation analysis is a valuable method for assessing the most cited articles. Local citation frequency is used to reflect the impact of articles in a particular research field. The top three cited journals were Annals of Surgery (926 co-citations), British Journal of Surgery (574 co-citations), and British Journal of Anaesthesia (556 co-citations). Furthermore, a co-citation analysis of references was carried out. The minimum number of citations was set as 35. A total of 49 studies were obtained to draw the co-citation map (Fig. 5). More than 70% of the articles in the green cluster were published before 2010, representing the early development stage of this field. Most studies in the green cluster focused on orthopedic surgery, from which we could learn the origin of the concept of prehabilitation (Topp et al. 2002; Carli and Zavorsky 2005). The literature in the red cluster was published from 2010 to 2017, and the research subject was transferred to abdominal surgery, especially colorectal cancer surgery. The blue cluster was the articles published in the last 5 years. Fifty percent of the articles were meta-analyses and systematic reviews, which comprehensively summarized the conclusions of previous studies and provided more reliable clinical evidence. The others were clinical studies with a shift in content, which focused on elderly, frail patients, patient adherence, and patient-reported outcomes (PROs). Among the top 10 cited articles, 1 article was published in 2018. The remaining 9 articles were all published before 2016, including 6 clinical trials, 2 meta-analyses, and 1 review (Supplementary Table S1). To account for the effect of publication time on citations, we also counted the top 10 cited articles on average annual citations (Supplementary Table S2). The 2 articles were duplicated with Supplementary Table S1 and 8 new articles published from 2018 to 2022 were additionally retrieved.

## Discussion

This bibliometric study analyzed studies related to the rehabilitation of surgical patients in the past 20 years in terms of publication trends, cooperation networks, study themes, and co-citation relationships. Prehabilitation is a new research direction. We found that the number of publications in this field began to explode in 2016, and 70% of articles were published in the past 5 years (2019–2023). This phenomenon may be related to the promotion of ERAS. As one of the important aspects of ERAS, rehabilitation has been recommended by clinical guidelines (Batchelor et al. 2019; Gustafsson et al. 2019; Hübner et al. 2020). Moreover, the growth trend of publications related to prehabilitation was similar to that of previous ERAS-related studies (Li et al. 2021). In a word, according to the growth trend of publications, articles published in high-impact journals, and multiple registered clinical study protocols (Rooijen et al. 2019; García-Delgado et al. 2021; Santa Mina et al. 2021), research related to prehabilitation is still ongoing and deserves continuous attention.

The cooperative network analysis showed that clinical experts from high-income countries in North America and Europe were the main contributors to the research in this field, which may be inextricably linked to the size of the population of these countries, but also their strong and mature research capabilities. The analysis provides unique bibliometric information for researchers in different countries to determine the contribution of their regions in this research field. Although research collaboration among countries had been formed, regional limitations still existed. Links between author groups were also relatively sparse, suggesting that international collaboration was not well established. The first international multicenter study protocol of prehabilitation was published in 2019 and involved centers from the Netherlands, Canada, Denmark, France, Italy, and Spain (Rooijen et al. 2019), but without low- and middle-income countries. Collaboration between researchers from different countries should be enhanced.

The number of citations is a commonly used index to evaluate the academic value and influence of articles in bibliometric analysis (Cooper 2015). A higher number of citations means that the information provided by the article is helpful to researchers and reflects the high value of the article. This study found that almost all the top 10 articles by total citations were published before 2016. The high total citation of the article indicated that the results were significant, which provided ideas and references for further study. However, the publication time of the article was also a non-ignorable factor, which the early publication time helped to accumulate the number of citations. Therefore, we further conducted an annual average analysis of total citations. The result showed that 7 of the top 10 articles were published in the last 5 years. The two lists do not contradict each other. We should not dismiss the value of articles published within the last 5 years solely based on the total citations. Newly published articles with high average annual citations could also provide important information to readers. Most of these high-cited articles focused on the effect of prehabilitation on patients undergoing elective abdominal surgery, especially colorectal cancer surgery, which is consistent with the results of keyword analysis. The possible reason was that the ERAS strategy was first implemented in this type of surgery and achieved good effects (Lassen et al. 2009).In addition, most of the high-cited articles were RCT and meta-analysis, which also indicated that articles with high levels of evidence quality would receive more attention.

A number of high-cited articles summarized in our study found that prehabilitation could promote the recovery of exercise capacity in patients with abdominal cancer after surgery, but did not reduce complications, length of hospital stays, or improve the quality of postoperative recovery (Gillis et al. 2014; Dronkers et al. 2010; Li et al. 2013; Minnella et al. 2018). Given that the above study enrolled low-risk patients, subsequent studies focused on elderly or frail patients. Barberan-Garcia et al. found that prehabilitation reduced the incidence of complications in elderly patients undergoing major abdominal surgery (Barberan-Garcia et al. 2018). However, an RCT completed by Carli et al. suggested that multimodal prehabilitation did not improve postoperative outcomes in frail patients undergoing colorectal cancer surgery, including length of hospital stay, complications, and readmission rates (Carli et al. 2020). Limited by the quality and heterogeneity of different studies, meta-analyses on the effects of prehabilitation in patients undergoing abdominal surgery failed to provide convincing evidence pertaining to complications, length of hospital stay, and mortality (Moran et al. 2016b; Hughes et al. 2019). The conflicting results between different studies suggested that there still existed space for further optimization of prehabilitation protocols and verifying its effects on the elderly, such as improving patient compliance with prehabilitation strategies (Bruns et al. 2016; Ripollés-Melchor et al. 2022), using wireless wearable devices for monitoring (Strath and Rowley 2018), using more sensitive patient-reported outcomes to evaluate the effect of interventions (Warnakulasuriya et al. 2020), and strengthening the doctor’s supervision and guidance to patients (Beck et al. 2022).

This paper has some limitations. First, although WOSCC is a large and reliable database for bibliometric analysis (Falagas et al. 2008), other English and non-English databases may contain more potential papers. Second, the search keywords related to prehabilitation were quite general, including exercise, psychological intervention, and nutrition. The understanding and definition of prehabilitation may be different among different researchers. Our search formula may miss some studies related to the prehabilitation of surgical patients. Third, some keywords appeared frequently, but they do not contain useful information (e.g., review, research, and meta-analysis) and cannot be analyzed. Future research can broaden the search scope, improve the search formula, and explore more relevant studies.

## Conclusion

We summarized the progress and future directions of prehabilitation of surgical patients through bibliometric analysis. This field has great research prospects. Researchers from North America and European countries have made outstanding contributions in this field. Abdominal surgery, especially colorectal cancer surgery, has been extensively researched. Malnutrition, older patients, frailty, and high-risk patients are the hotspots of recent studies. However, the results of different studies are not consistent, and further study is still needed to fill the current deficiencies.

### Supplementary Information

Additional file 1: Supplementary Figure S1. Screening flow chart. Supplementary Figure S2. Cooperation relationship between top 20 countries with most publications. Supplementary Figure S3. Density map based on publication numbers of authors. Supplementary Table S1. Top 10 cited references. Supplementary Table S2. Top 10 references by average annual citations.

## Data Availability

The datasets used and/or analysed during the current study are available from the corresponding author on reasonable request.

## Abbreviations

ERAS: Enhanced recovery after surgery
WOSCC: Web of Science Core Collection
SCI-expanded: Science Citation Index Expanded
SSCI: Social Sciences Citation Index
JCR: Journal Citation Reports
6MWD: 6-Minute walking distance
PROs: Patient-reported outcomes
RCTs: Randomized controlled trials
UK: The United Kingdom
USA: The United States of America

## References

[CR1] Barberan-Garcia A, Ubré M, Roca J (2018). Personalised prehabilitation in high-risk patients undergoing elective major abdominal surgery: a randomized blinded controlled trial. Ann Surg.

[CR2] Batchelor TJP, Rasburn NJ, Abdelnour-Berchtold E (2019). Guidelines for enhanced recovery after lung surgery: recommendations of the Enhanced Recovery After Surgery (ERAS®) Society and the European Society of Thoracic Surgeons (ESTS). Eur J Cardiothorac Surg.

[CR3] Beck A, Thaysen HV, Soegaard CH (2022). Investigating the experiences, thoughts, and feelings underlying and influencing prehabilitation among cancer patients: a qualitative perspective on the what, when, where, who, and why. Disabil Rehabil.

[CR4] Bruns ER, van den Heuvel B, Buskens CJ (2016). The effects of physical prehabilitation in elderly patients undergoing colorectal surgery: a systematic review. Colorectal Dis.

[CR5] Carli F (2020). Prehabilitation for the Anesthesiologist. Anesthesiology.

[CR6] Carli F, Zavorsky GS (2005). Optimizing functional exercise capacity in the elderly surgical population. Curr Opin Clin Nutr Metab Care.

[CR7] Carli F, Bousquet-Dion G, Awasthi R (2020). Effect of multimodal prehabilitation vs postoperative rehabilitation on 30-day postoperative complications for frail patients undergoing resection of colorectal cancer: a randomized clinical trial. JAMA Surg.

[CR8] Cooper ID (2015). Bibliometrics basics. J Med Libr Assoc.

[CR9] Cusack B, Buggy DJ (2020). Anaesthesia, analgesia, and the surgical stress response. BJA Educ.

[CR10] Derviş H (2019). Bibliometric analysis using Bibliometrix an R Package. J Scientometric Res.

[CR11] Donthu N, Kumar S, Mukherjee D, Pandey N, Lim WM (2021). How to conduct a bibliometric analysis: an overview and guidelines. J Bus Res.

[CR12] Dronkers JJ, Lamberts H, Reutelingsperger IM (2010). Preoperative therapeutic programme for elderly patients scheduled for elective abdominal oncological surgery: a randomized controlled pilot study. Clin Rehabil.

[CR13] Falagas ME, Pitsouni EI, Malietzis GA, Pappas G (2008). Comparison of PubMed, Scopus, Web of Science, and Google Scholar: strengths and weaknesses. Faseb j.

[CR14] García-Delgado Y, López-Madrazo-Hernández MJ, Alvarado-Martel D (2021). Prehabilitation for bariatric surgery: a randomized, controlled trial protocol and pilot study. Nutrients..

[CR15] Gillis C, Li C, Lee L (2014). Prehabilitation versus rehabilitation: a randomized control trial in patients undergoing colorectal resection for cancer. Anesthesiology.

[CR16] Gillis C, Ljungqvist O, Carli F (2022). Prehabilitation, enhanced recovery after surgery, or both? A narrative review. Br J Anaesth.

[CR17] Gustafsson UO, Scott MJ, Hubner M (2019). Guidelines for perioperative care in elective colorectal surgery: enhanced recovery after surgery (ERAS(®)) society recommendations: 2018. World J Surg.

[CR18] Hübner M, Kusamura S, Villeneuve L (2020). Guidelines for Perioperative Care in Cytoreductive Surgery (CRS) with or without hyperthermic IntraPEritoneal chemotherapy (HIPEC): Enhanced recovery after surgery (ERAS®) Society Recommendations - Part I: Preoperative and intraoperative management. Eur J Surg Oncol.

[CR19] Hughes MJ, Hackney RJ, Lamb PJ, Wigmore SJ, Christopher Deans DA, Skipworth RJE (2019). Prehabilitation before major abdominal surgery: a systematic review and meta-analysis. World J Surg.

[CR20] Kehlet H (2021). Prehabilitation in surgery - need for new strategies?. Eur J Surg Oncol.

[CR21] Lassen K, Soop M, Nygren J (2009). Consensus review of optimal perioperative care in colorectal surgery: Enhanced Recovery After Surgery (ERAS) Group recommendations. Arch Surg.

[CR22] Li C, Carli F, Lee L (2013). Impact of a trimodal prehabilitation program on functional recovery after colorectal cancer surgery: a pilot study. Surg Endosc.

[CR23] Li C, Cheng Y, Li Z, Margaryan D, Perka C, Trampuz A (2021). The pertinent literature of enhanced recovery after surgery programs: a bibliometric approach. Medicina (Kaunas)..

[CR24] Minnella EM, Awasthi R, Loiselle S-E, Agnihotram RV, Ferri LE, Carli F (2018). Effect of exercise and nutrition prehabilitation on functional capacity in esophagogastric cancer surgery: a randomized clinical trial. JAMA Surg.

[CR25] Moran J, Wilson F, Guinan E, McCormick P, Hussey J, Moriarty J (2016). Role of cardiopulmonary exercise testing as a risk-assessment method in patients undergoing intra-abdominal surgery: a systematic review. Br J Anaesth.

[CR26] Moran J, Guinan E, McCormick P (2016). The ability of prehabilitation to influence postoperative outcome after intra-abdominal operation: a systematic review and meta-analysis. Surgery.

[CR27] Orri M, Boleslawski E, Regimbeau JM (2015). Influence of depression on recovery after major noncardiac surgery: a prospective cohort Study. Ann Surg..

[CR28] Ripollés-Melchor J, Abad-Motos A, Cecconi M (2022). Association between use of enhanced recovery after surgery protocols and postoperative complications in colorectal surgery in Europe: the EuroPOWER international observational study. J Clin Anesth.

[CR29] Santa Mina D, Sellers D, Au D (2021). A pragmatic non-randomized trial of prehabilitation prior to cancer surgery: study protocol and COVID-19-related adaptations. Front Oncol.

[CR30] Strath SJ, Rowley TW (2018). Wearables for promoting physical activity. Clin Chem.

[CR31] Topp R, Ditmyer M, King K, Doherty K, Hornyak J (2002). The effect of bed rest and potential of prehabilitation on patients in the intensive care unit. AACN Clin Issues.

[CR32] Van Eck N, Waltman L (2010). Software survey: VOSviewer, a computer program for bibliometric mapping. Scientometrics..

[CR33] van Rooijen S, Carli F, Dalton S (2019). Multimodal prehabilitation in colorectal cancer patients to improve functional capacity and reduce postoperative complications: the first international randomized controlled trial for multimodal prehabilitation. BMC Cancer.

[CR34] Warnakulasuriya SR, Patel RC, Singleton GF, Moonesinghe SR (2020). Patient-reported outcomes for ambulatory surgery. Curr Opin Anaesthesiol.

[CR35] Weiser TG, Haynes AB, Molina G (2015). Estimate of the global volume of surgery in 2012: an assessment supporting improved health outcomes. Lancet.

[CR36] Williams JB, Alexander KP, Morin JF (2013). Preoperative anxiety as a predictor of mortality and major morbidity in patients aged >70 years undergoing cardiac surgery. Am J Cardiol.

[CR37] Wischmeyer PE, Carli F, Evans DC (2018). American society for enhanced recovery and perioperative quality initiative joint consensus statement on nutrition screening and therapy within a surgical enhanced recovery pathway. Anesth Analg.

