# Supplementary material for: Prehabilitation of surgical patients: a bibliometric analysis from 2005 to 2023
Source: Perioper Med (Lond). 2024 May 31;13:48. doi: 10.1186/s13741-024-00410-x (PMC11140917; doi:10.1186/s13741-024-00410-x)
Supplement: Supplementary file 1 — Additional file 1: Supplementary Figure S1. Screening flow chart. Supplementary Figure S2. Cooperation relationship between top 20 countries with most publications. Supplementary Figure S3. Density map based on publication numbers of authors. Supplementary Table S1. Top 10 cited references. Supplementary Table S2. Top 10 references by average annual citations. [file 13741_2024_410_MOESM1_ESM.docx]

**Supplementary materials**

**Supplementary Figure 1.** Screening flow chart.

Supplementary Figure 2. Cooperation relationship between top 20 countries with most publications.


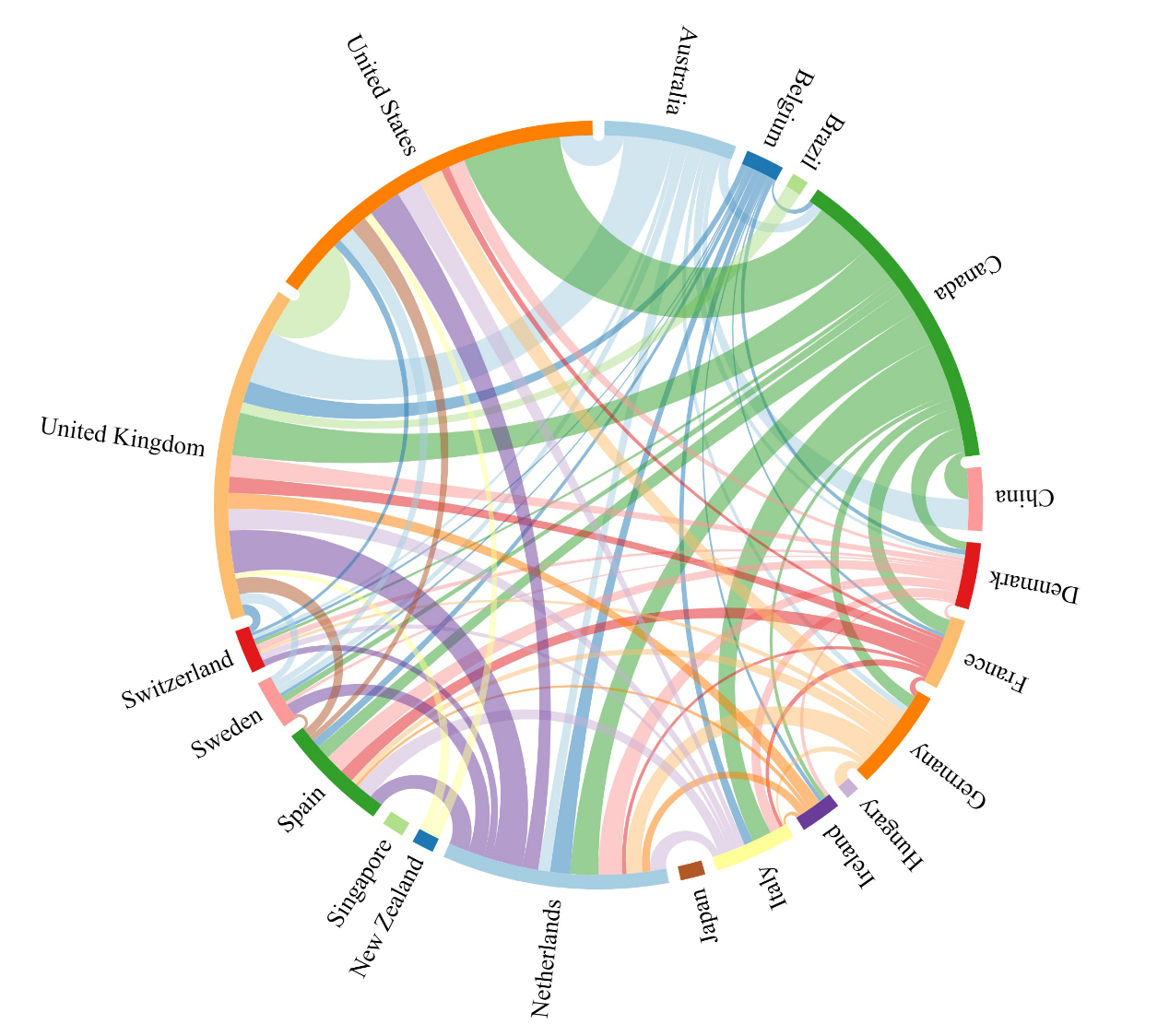


Supplementary Figure 3. Density map based on publication numbers of authors.


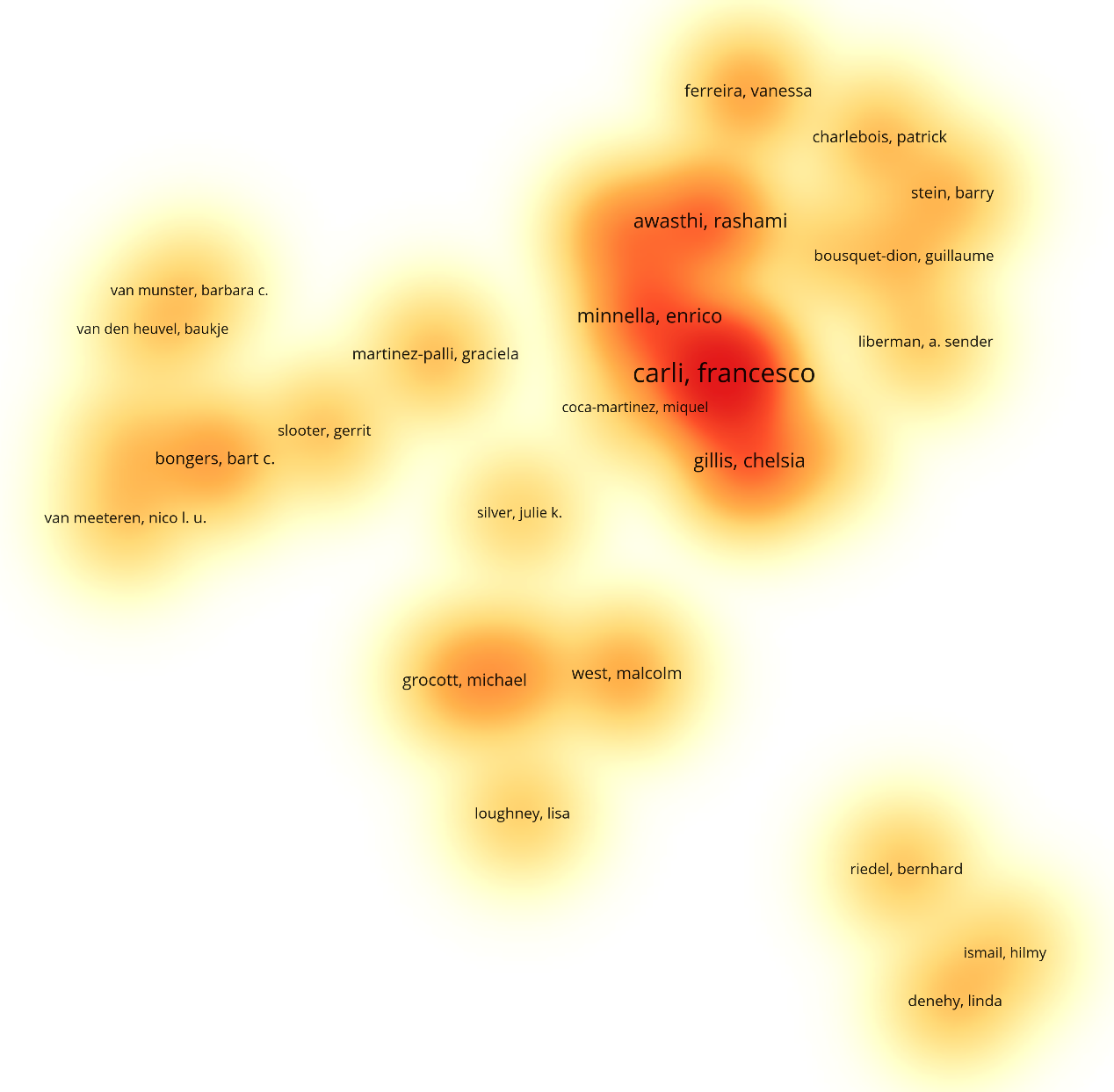


| **Title** | **Article type** | **Year** | **Journal/JCR** | **Corresponding Author** | **Local citations** |
| --- | --- | --- | --- | --- | --- |
| Prehabilitation versus rehabilitation: a randomized control trial in patients undergoing colorectal resection for cancer | RCT | 2014 | Anesthesiology/Q1 | Francesco Carli | 176 |
| Personalised Prehabilitation in High-risk Patients Undergoing Elective Major Abdominal Surgery: A Randomized Blinded Controlled Trial | RCT | 2018 | Annals of surgery/Q1 | Anael Barberan-Garcia | 148 |
| Impact of a trimodal prehabilitation program on functional recovery after colorectal cancer surgery: a pilot study | RCT | 2013 | Surgical endoscopy/Q1 | Liane S. Feldman | 132 |
| Randomized clinical trial of prehabilitation in colorectal surgery | RCT | 2010 | British journal of surgery/Q1 | Francesco Carli | 114 |
| Effect of prehabilitation on objectively measured physical fitness after neoadjuvant treatment in preoperative rectal cancer patients: a blinded interventional pilot study | Nonrandomized control trial | 2015 | British journal of anaesthesia/Q1 | M. A. West | 94 |
| The ability of prehabilitation to influence postoperative outcome after intra-abdominal operation: A systematic review and meta-analysis | Meta-analysis | 2016 | Surgery/Q1 | Jonathan Moran | 89 |
| Optimizing functional exercise capacity in the elderly surgical population | Review | 2005 | Current opinion in clinical nutrition and metabolic care/Q3 | Francesco Carli | 87 |
| Effect of total-body prehabilitation on postoperative outcomes: a systematic review and meta-analysis | Meta-analysis | 2014 | Physiotherapy/Q1 | D. Santa Mina | 82 |
| Classification of surgical complications: a new proposal with evaluation in a cohort of 6336 patients and results of a survey | Cross-sectional study | 2004 | Annals of surgery/Q1 | Pierre-Alain Clavien | 80 |
| Preoperative therapeutic programme for elderly patients scheduled for elective abdominal oncological surgery: a randomized controlled pilot study | RCT | 2010 | Clinical rehabilitation/Q1 | J J Dronkers | 80 |

Supplementary Table 1. Top 10 cited references

JCR: Journal citation report; RCT: Randomized control trial

Supplementary Table 2. Top 10 references by average annual citations

| **Title** | **Article type** | **Year** | **Journal/Year** | **Corresponding Author** | **Average annual citations** |
| --- | --- | --- | --- | --- | --- |
| Effects of Community-based Exercise Prehabilitation for Patients Scheduled for Colorectal Surgery With High Risk for Postoperative Complications: Results of a Randomized Clinical Trial | RCT | 2022 | Annals of surgery/Q1 | Joost M. Klaase | 41.0 |
| Personalised Prehabilitation in High-risk Patients Undergoing Elective Major Abdominal Surgery: A Randomized Blinded Controlled Trial | RCT | 2018 | Annals of surgery/Q1 | Anael Barberan-Garcia | 29.6 |
| Effect of Multimodal Prehabilitation vs Postoperative Rehabilitation on 30-Day Postoperative Complications for Frail Patients Undergoing Resection of Colorectal Cancer: A Randomized Clinical Trial | RCT | 2020 | JAMA surgery/Q1 | Francesco Carli | 22.7 |
| Prehabilitation versus rehabilitation: a randomized control trial in patients undergoing colorectal resection for cancer | RCT | 2014 | Anesthesiology/Q1 | Francesco Carli | 20.0 |
| Prehabilitation in adult patients undergoing surgery: an umbrella review of systematic reviews | Review | 2022 | British journal of anaesthesia/Q1 | Daniel I. McIsaac | 19.0 |
| Prehabilitation Before Major Abdominal Surgery: A Systematic Review and Meta-analysis | Meta-analysis | 2019 | World journal of surgery/Q2 | Michael J. Hughes | 16.5 |
| Effects of Nutritional Prehabilitation, With and Without Exercise, on Outcomes of Patients Who Undergo Colorectal Surgery: A Systematic Review and Meta-analysis | Meta-analysis | 2018 | Gastroenterology/Q1 | Chelsia Gillis | 15.6 |
| Investigating the experiences, thoughts, and feelings underlying and influencing prehabilitation among cancer patients: a qualitative perspective on the what, when, where, who, and why | Cross-sectional study | 2022 | Disability and rehabilitation/Q2 | Anne Beck | 15 |
| Multimodal Prehabilitation to Enhance Functional Capacity Following Radical Cystectomy: A Randomized Controlled Trial | RCT | 2021 | European urology focus/Q1 | Enrico M. Minnella | 14 |
| Multimodal prehabilitation in colorectal cancer patients to improve functional capacity and reduce postoperative complications: the first international randomized controlled trial for multimodal prehabilitation | Protocol | 2019 | BMC cancer/Q2 | Gerrit Slooter | 13.5 |

RCT: Randomized control trial
